# Treatment efficacy of ^153^Sm-EDTMP for painful bone metastasis

**DOI:** 10.7508/aojnmb.2013.01.006

**Published:** 2013

**Authors:** Narjess Ayati, Kamran Aryana, Amir Jalilian, Toktam Hoseinnejad, Ali Bahrami Samani, Zahra Ayati, Farzane Shariati, S. Rasoul Zakavi

**Affiliations:** 1 Nuclear Medicine Research Center, Mashhad University of Medical Science, Faculty of Medicine, Mashhad, Iran; 2 Radiopharmaceutical Research & Development Laboratory (RRDL), Nuclear Science and Technology Research Institute (NSTRI), Tehran, Iran; 3 School of pharmacy, Mashhad University of Medical Science, Mashhad, Iran

**Keywords:** Radionuclide Therapy, Samarium, 153Sm-EDTMP, painful bone metastasis

## Abstract

**Introduction:**

Involvement of the skeleton can cause an excruciating pain in two-thirds of terminal patients with a history of malignancy. Due to several limitations of other therapies, such as analgesics, bisphosphonates, chemotherapy, hormonal therapy and external beam radiotherapy; bone-seeking radiopharmaceuticals have an important role in palliation of pain from bone metastases. Although these kinds of therapies have many advantages including the ability to treat multiple sites of tumoral involvement simultaneously, no significant confliction with other treatments, ease of administration and the potential to be used repetitively; in Iran using of this modality is not widely practiced. In this study we evaluated the clinical usefulness of Sm-153 lexidronamfor pain management of bone metastases.

**Methods:**

28 patients (14 males and 14 females) aged 38-77 years with a history of painful bone metastases caused by different cancers, not responding to conventional treatments were included in the study. All patients had a recent whole body bone scan indicating multiple bone metastases. 1 mCi/Kg Sm-153 lexidronam was injected intravenously to the patients. Whole body scintigraphy was done 3 or 18 hours post injection. Pain relief and quality of life have been evaluated by analog pain scale and Karnofsky index every week, respectively. Also, all patients were evaluated for hematological toxicity every two weeks. Active follow ups were performed.

**Results:**

43% of patients showed the presence of the flare phenomenon during the first three days after Sm injection with a mean duration of 2.2 days. The pain relief began between 2 and 16 days post injection and the duration of pain palliation was in the range of 4 to 32 weeks (mean±SD=15.22±7.8). 64.3% of patients showed complete relief of pain and 21.4% achieved partial response to therapy. (Over all response to therapy was 85.7%). The lowest amount of peripheral blood cells was detected in the fourth week for RBCs and in the 6th week for WBCs and PLTs. No one experienced hematological toxicity induced problems.

**Conclusion:**

Sm-153 lexidronam is an effective treatment for painful bone metastases. The complication rate is low and the quality of life of the patients after treatment would be significantly improved.

## Introduction

Bone metastasis is the common consequence of many malignancies including prostate, breast and lung cancers. One of the most unfavorable complications of metastatic bone involvement is skeletal pain, which can be seen in two-thirds of terminal patients(1). The pain can be highly excruciating and intolerable which significantly affects the life quality of the patients. Multidisciplinary approach to this complication has received a lot of attention resulting in different kinds of treatments but each of them has its own drawbacks which limit their usage.

Generally usage of non-steroidal anti-inflammatory drugs (NSAIDs) is the first step towards pain relief, which may be replaced by narcotics with the progression of the disease. Narcotic usage itself is commonly accompanied by complications such as over sedation, constipation and severe nausea which strongly reduce the life quality of the patients. Other systemic treatments such as chemotherapy and hormonal therapy have their own limitations as well. On the other hand, some treatment modalities such as external beam radiotherapy and surgery, because of their localized nature, are not optimal solutions for patients with widespread bone metastasis.

Bone pain palliation using beta emitter radioisotopes plays a pivotal role in patients with osteoblastic or mixed bone metastasis. This kind of therapy has some unique advantages including the potential ability for simultaneous pain relief in multiple sites of bone involvement, no confliction with other treatments, facility of administration and the potential to be used repetitively. Despite these valuable attributes, unfortunately in Iran use of this method has been very limited.

Samarium-153 Ethylene Diamine Tetra Methylene Phosphonate (^153^Sm-EDTMP) is one of the most practical radiopharmaceuticals for bone pain palliation with the ability to concentrate in the areas with increased osteoblastic activity. As Samarium emits both gamma and beta particles, it is possible to confirm bone accumulation of the radiotracer after administration. The average range of its beta rays is about 6 millimeters in soft tissue and its physical half life is nearly 46 hours. Reported response rate of ^153^Sm-EDTMP administration is up to 92%(2) with associated significant reduction in costs of patient’s cancer management(3). The major complication of radionuclide therapy (RnT) is self limiting bone marrow toxicity(4-5). Current new trends for increasing the effectiveness of this modality is using a combination of RnT with chemotherapy and administration of two different kinds of radiotracers with a short duration interval(6-8).

The aim of this study was evaluating the effectiveness of ^153^Sm-EDTMP on bone pain palliation as well as a precise assessment of its negative and positive consequences.

## Methods

28 consecutive patients (14 males) aged 38 to 77 years (mean age ± SD=59±12) who had painful skeletal metastases were included in the study. Each patient had at least two sites of severe bone pain due to osteoblastic or mixed bone metastases (according to a recent bone scan) with no evidence of any contraindications for radionuclide therapy. The exclusion criteria included pregnancy and breast feeding, any evidence of Disseminated Intravascular Coagulation (DIC), super scan pattern in the latest bone scintigraphy, any emergency problems such as acute compression on the spinal cord and pathologic fractures, neurologic origin as the source of bone pain, hemi body radiotherapy during the last three months and long acting chemotherapy during the last four weeks. After taking a comprehensive medical history and giving written and oral instructions to patients regarding the treatment and suitable hydration, ^153^Sm-EDTMP (produced by Radiopharmaceutical Research & Development Laboratory (RRDL), Nuclear Science and Technology Research Institute (NSTRI), Tehran, Iran) with the dose of 1mCi/kg was injected intravenously followed by rapid infusion of 10cc saline 0.9%.

The patients were observed in the first 4 hours post injection for appropriate hydration, control of symptoms and social radiation protection. Before injection of ^153^Sm-EDTMP, urinary catheter was inserted for the patients with urinary incontinence and remained in place for 24 hours. Whole body scan was performed 3 and/or 18 hours after ^153^Sm-EDTMP administration for evaluation of drug distribution and confirmation of radiotracer accumulation in the lesions.

The patients were followed up every three days during the first two weeks and then every week for the next 16 weeks. The severity of bone pain (by visual analog scale (VAS) ruler(9)), as well as general health status including the quality of sleep, appetite, social interaction and the amount of analgesic consumption were recorded. The patient’s performance status was evaluated by two separate criteria (Karnofsky(10) and ECOG(11)) before ^153^Sm-EDTMP injection and then every week post treatment.

Therapeutic response was defined as pain palliation after samarium injection based on the VAS ruler. The VAS scores less than 3 points after treatment was considered a complete response. Delta VAS (∆VAS) was defined as the difference between pre-treatment and post treatment VAS scores. No response was defined as ∆VAS less than 3(Negligible pain palliation). Other conditions were considered as partial response.

Analgesic usage was categorized in to two groups; non-steroidal anti inflammatory agents (NSAIDs) and narcotic drugs and the average usage of both was recorded before and after the treatment. In our study, the number of NSAIDs was seen equivalent to the same number of Ibuprofen tablets (400 mg) and the narcotic drugs were considered equivalent to 10 mg of morphine in intra muscular injection form.

Assessment of bone marrow function was done every 2 weeks by evaluation of peripheral blood cell counts for 12 weeks and the grade of bone marrow toxicity was obtained based on the National Cancer Institute Common Toxicity Criteria (NCI-CTC).

The data was finally analyzed by the SPSS 16 software. Univariate analysis was done for description of frequency of different variables. Comparison of quantitative variables before and after therapy was done using a paired t-test.

## Results

43% (12 out of 28) of the cases had breast cancer, 29% (8 cases) prostate cancer, 11% nasopharyngeal carcinoma and 5 cases (17%) were involved with one of the following; lung, rectum, bladder or gastric carcinomas. The treatment was explained to the patients and then an informed written consent was obtained. All patients suffered severe pain in at least two points of the skeleton and 75% of them (21 patients) had severe bone pain in three or more foci. Consistent with the VAS ruler, the mean severity of bone pain was 9.75 in the first site, 9.10 in the second site and 8.66 in the third site of the skeleton.

Bone pain intensified in 43% of cases between the first and the third days post injection with the mean duration of 2.2 days (Mean±SD =2.2±0.83) and was considered as the flare phenomenon.

Pain relief began between 2 to 16 days post injection (Mean±SD =8.4±4.3) and the response lasted for 4 to 32 weeks with a mean duration of 15.2 weeks. (Mean±SD =15.2±7.8). Complete and partial response was observed in 18 and 6 cases (64.3% & 21.4%) respectively and 4 cases (14.3%) had no response to therapy.

**Figure 1:**
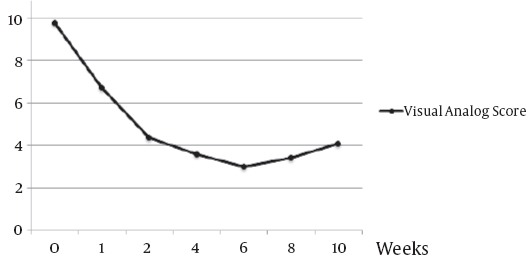
The mean pain severity (VAS) before and after 153Sm-EDTMP administration

Figure 1 shows the mean pain severity before and after treatment.

There was no significant difference between patients with breast, prostate & others cancers in terms of treatment response. (p=0.118)

The general health status improved in 46% of patients, 25% reported no change and 29% had worse condition post ^153^Sm-EDTMP injection.

Table 1 shows the effect of treatment on patient’s performance status based on ECOG and Karnofsky criteria. (Table 1)

**Table 1:**
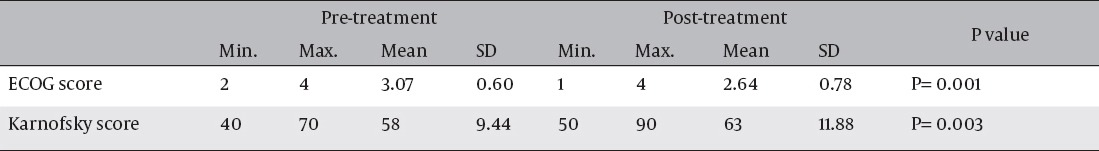
Comparison of patients’ performance status pre and post 153Sm-EDTMP administration

**Figure 2:**
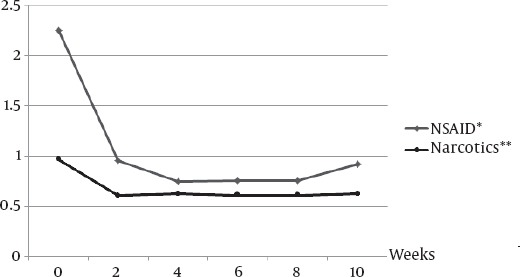
The average of analgesic usage before and post 153Sm-EDTMP administration * The amount of NSAID consumption has been calculated based on the equivalent dose of 400 mg Ibuprofen (PO) ** The amount of Narcotic consumption has been calculated based on the equivalent dose of 10 mg Morphine (IM)

The amount of analgesic consumption was significantly reduced post injection (p<0.001). Figure 2 shows the average of narcotic & NSAID usage before and during the 12 week post treatment. (Figure 2)

**Figure 3:**
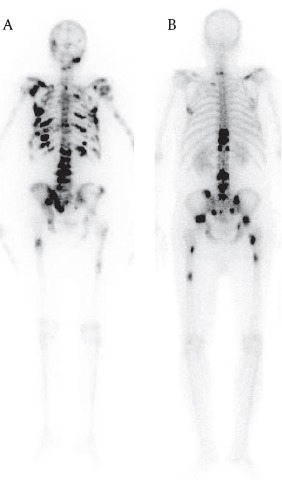
Whole body scans in posterior views 3 (A) and 18 hours (B) post 153Sm-EDTMP administration in two different patients with breast (A) and prostate (B) carcinomas

Whole body scans done 3 and/or 18 hours post injection were both of good quality and in a one-by-one comparison, all hot zones of tumoral involvement in the recent bone scans had increased ^153^Sm-EDTMP uptake in the following scintigraphies. No abnormal soft tissue uptake was noted in samarium scans. (Figure 3)

Although WBC and PLT dropped earlier than RBC (Table 2), significant reduction in RBC counts occurs four weeks post injection (p=0.02) and significant reduction in WBC and PLT counts was seen six weeks after RnT (p=0.008 & p=0.01). No one showed grade 4 bone marrow toxicity. Grade 3 was observed in 4 cases (14.2%) for white blood cells and in 3 cases (10.7%) for platelets. All other cases were between grades 0 to 2 for bone marrow toxicity. The mean blood cells counts, before and after treatment, are shown in table 2. The mean blood cell count 10 weeks post ^153^Sm-EDTMP injection was not significantly different from the pre-treatment count.

**Table 2:**
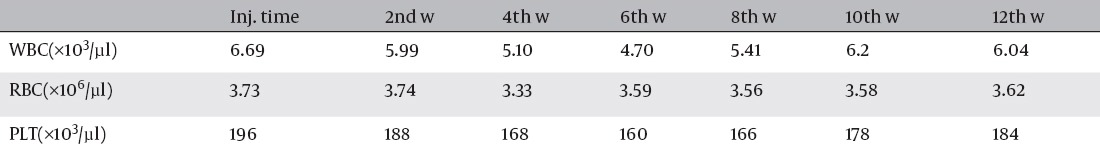
The mean blood cell count before and after 153Sm-EDTMP administration

## Discussion

Radionuclide Therapy (RnT) is a well known universal treatment for patients with severe metastatic bone pain(5-6).

Until now, a wide range of response rate has been reported by different clinical trials. This discrepancy can be due to the usage of different kinds of radiotracers, different criteria for response assessment and possibly the variety of inclusion criteria (kind of malignancy, stage of disease at the time of RnT and so on). A large well-designed review showed a pooled range of 70-90% response rate with 30-40% complete response according to the previous studies.(5) Two important studies which have evaluated the efficacy of ^153^Sm-EDTMP on metastatic bone pain due to breast and prostate cancers specifically, showed 81 and 92% over all response rate with 51 and 36.5% complete response respectively(2,12). Two other retrospective studies which evaluated the clinical role of ^153^Sm-EDTMP on a large group of patients with different kinds of malignancies showed 85 and 80% total response and 21 and 40% complete response respectively(13-14). Based on this report, the median time of improvement onset was 7 days after ^153^Sm-EDTMP injection with the mean effect duration of 3 months. In our study complete response was achieved in 13 cases (46%); however 39% also showed some degrees of pain relief. This results in an 85% over all response. Also the duration of pain palliation by RnT showed a wide range (4 to 32 weeks) which makes the prediction of response duration difficult. Although our patients showed a higher rate of complete response compared to the study of Baczyket al (14), the overall response as well as the onset and duration of pain relief was compatible.

As there was a possibility that the patients continued narcotic usage despite the pain palliation, we evaluated NSAIDs and narcotic usage separately. Interestingly, both pharmacologic groups showed significant reduction in usage following the onset of decrease in patients VAS scores.

The study showed significant reduction in all three types of blood cell counts (WBC, RBC and PLT), but the RBC reduction had some differences with WBCs and PLTs in both severity and time of onset. Grade 3 bone marrow toxicity was not observed for RBCs. This suggests more concern for WBCs and PLTs. At ten weeks post treatment the blood cell count was similar to the pre-treatment count although there are some studies which suggest longer time for blood cell count reversibility(15).

Absence of life threatening events as a result of ^153^Sm-EDTPM injection makes administration of this radiotracer more favorable. In this aspect, our results were congruent with the results of other similar studies(16-18).

We faced some limitations during our study. First of all, our study was limited to only 28 cases; therefore, sub groups analysis was not possible. We suggest further clinical trials with larger groups of patients in each kind of malignancy. Secondly, evaluation of specific serum markers and follow up bone scintigraphy were not done post treatment, so the response assessment was based only on patient’s reports.

## Conclusion

Samarium-EDTMP therapy is an effective and safe solution for metastatic bone pain palliation with no fatal complications. We hope this favorable therapy becomes a common and frequent procedure in Iran in the near future.
